# Fast Dynamics of Cortical Functional and Effective Connectivity during Word Reading

**DOI:** 10.1371/journal.pone.0088940

**Published:** 2014-02-14

**Authors:** Nicolas Bedo, Urs Ribary, Lawrence M. Ward

**Affiliations:** 1 Department of Psychology, University of British Columbia, Vancouver, British Columbia, Canada; 2 Behavioral and Cognitive Neuroscience Institute, Simon Fraser University, Burnaby, British Columbia, Canada; 3 Department of Psychology, Simon Fraser University, Burnaby, British Columbia, Canada; 4 Brain Research Centre, University of British Columbia, Vancouver, British Columbia, Canada; Cuban Neuroscience Center, Cuba

## Abstract

We describe for the first time the fast dynamics of functional and effective (causal) connectivity during word reading. Independent component analysis of high-density EEG recorded during a word reading task recovered multiple sources of electrical brain activity previously identified by fMRI and PET. Results confirmed the ventral occipito-temporal cortex (vOT) as a central hub for word reading, showing a progression of theta-band (3–7 Hz) and gamma-band (30–50 Hz) phase synchronization and directed theta-band and gamma-band information flow with both early visual areas and high-level language-processing areas. These results highlight the interplay between local and long-distance neural dynamics involved at each stage of the reading process. Moreover, these measures of functional and causal connectivity dynamics may be used as a benchmark for comparison with clinical populations (e.g. individuals with developmental dyslexia), such that disturbances in connectivity dynamics may provide insight as to underlying neurological problems with language processing, and their potential remediation.

## Introduction

Word reading is a multi-stage process that involves the extraction of information from orthographic symbols and requires the engagement of multiple visual, auditory, and language networks. This results in the emergence of complex and nuanced systems of semantics, pronunciation, grammar, and syntax. Efforts to determine how the brain processes orthography–and importantly, the impairments of such processing–have yielded useful frameworks from which to draw upon [1], [2]. As reading skill develops, established neural real estate is recycled and new brain networks are formed to support the task [3]. Even though much has been discovered with regard to mapping reading functions onto brain locations, however, the communication within and across these networks remains relatively unexplored.

A useful approach to examining networks is to investigate those brain areas that are critical for proper functioning. To that end, numerous studies have identified a region in left ventral occipito-temporal cortex (vOT), specifically in the left posterior fusiform gyrus, that is crucial to orthographic processing [4]. Dehaene and Cohen [3], among others, have championed the specialization of this region for orthographic processing, and named it the visual word form area (VWFA). The VWFA is nestled between basic visual and high-level language processing centers in the brain, affording it efficient access to these regions. A meta-analysis of 35 neuroimaging (fMRI and PET) studies further supported the notion of the VWFA as a central hub, crucial for processing orthographically-proper stimuli, that also relays visual word information to higher-level sites for further processing [5]. Moreover, a growing literature has shown white matter pathways linking the VWFA to brain areas related to reading, including occipital sites and language centers in the left hemisphere [6], [7].

The VWFA has been shown to respond preferentially to orthographic stimuli in specific ways. Neuroimaging studies have shown that consonant strings elicit activity in the VWFA, while checkerboards do not [8]. Additionally, it has been shown that the VWFA responds to written, but not to spoken, words [9]. Similarly, this region shows greater activation for words and word-like stimuli than for equally complex non-word-like stimuli [8]. Importantly, this activity is not dependent on the visual hemifield to which the word is presented [8]. That is, regardless of the location of the word in space, the information is routed to the VWFA in the left hemisphere for processing. Neuropsychological lesion studies show that damage to the VWFA may lead to deficits in reading performance, and perhaps even alexia, the inability to read [10], [11]. Event-related potential (ERP) studies have identified a reproducible negative peak in the ERP localized to the VWFA approximately 170 milliseconds (N170) exclusively after the presentation of a word [12]. The unilateral N170 ERP component generated by the VWFA is considered to be the standard measure of word form processing, and is used as a benchmark for ERP studies of word reading.

In their meta-analysis, Jobard and colleagues [5] identified higher-level brain areas responsible for word processing beyond the pre-lexical orthographic processing presumed to be accomplished by the VWFA. After basic word form processing, several cortical sites allow for semantic access, including left anterior [4]and posterior [13] middle temporal gyrus (MTG), left basal temporal areas, angular gyrus [4], [13] and left inferior frontal gyrus (IFG, pars triangularis) [14]. At the stage of grapho-phonological conversion–converting the word forms into sounds– left MTG and STG, left IFG (pars opercularis), and left supramarginal gyrus are engaged. Importantly, the details of the interactions among these areas during word reading, on a time scale of tens to hundreds of milliseconds, remain unclear.

The current understanding of how information is transmitted between brain regions during reading is minimal and comes primarily from inferences made by examining patterns of activation of localized sources rather than from direct measures of connectivity between those sources. For example, using MEG, Marinkovic et al [15] have described a pattern of activation starting in early visual cortex in the occipital lobe, which rapidly sweeps across the left hemisphere through the temporal lobe and ultimately arrives at language centers in the inferior frontal lobe. From results like these, it is clear that the reading signal is propagated from region to region. It remains unknown, however, just *how* these transitions occur across space, time, and oscillatory frequency, and how these brain areas are sharing information.

Some connectivity measures have been reported, however, offering a glimpse into the relations between some cortical sites during reading. Recently, Herdman [16] reported increased phase-locking of induced gamma-band (30+ Hz generally, 50–80 Hz reported here) oscillatory activity in posterior cortices during the perception of real letters compared with that during perception of pseudo-letters. This result suggests the existence of a preliminary orthographic evaluation network that determines whether or not visual symbol information is propagated to higher-level language centers. In an intracranial EEG study, Vidal et al [17] reported gamma amplitude correlations among reading-related sites, such as the left fusiform gyrus, IFG, MTG, superior temporal sulcus, and supramarginal gyrus. These correlations revealed potentially segregated semantic (ventral) and phonological (dorsal) networks, mirroring the dual-route model of word reading [1]. With regard to reading performance, Molinaro et al [18] posited that fronto-occipital theta-band (3–7 Hz) phase-locking supports the working memory aspects of sentence reading, aiding in the perception of expected words, while gamma-band phase-locking represents the evaluation of the orthographic symbols, further supporting claims by Herdman and Takai [19] that letter-processing is sensory-contingent.

Studies of white matter pathways show structural long-distance connections between visual and language areas [6], [7]. Exploring the dynamics of the functional and effective implications of these tracts may prove crucial to understanding reading in the brain more completely. Assuming that information integration and flow are implemented by synchronized oscillatory neural dynamics [20], it is likely that the study of such dynamics will reveal new important aspects of the word reading process.

We used high-density EEG to examine oscillatory dynamics, including functional and effective connectivity, in order to reveal information flow between brain regions during a word reading task. A series of hypotheses was tested. First, we tested the viability of independent component analysis (ICA) and subsequent single-dipole fitting to locate neural sources that have previously been identified by fMRI and PET studies. In this latter and all subsequent analyses we concentrated on regions of interest (ROI) in the left hemisphere of the brain, as those have proved to be the most robust for normal-reading right-handed participants, as ours were, and have been well-studied by other methods. Then, phase synchrony analysis was employed to calculate the degree of functional connectivity in specific oscillatory frequency bands between pairs of those cortical sites during word reading. We hypothesized that connectivity would follow established patterns of activation [15] spanning across the left hemisphere, and that this would occur in the theta (3–7 Hz) and gamma (30–50 Hz) frequency bands [17], [21], beginning in the posterior sensory cortices and gradually progressing to more anterior, high-level language centers. Additionally, it was expected that the vOT would play a crucial role as a central hub, linking low-level visual systems to specialized high-level language areas. Last, we measured narrow band transfer entropy in theta and gamma bands between IC activations localized to relevant brain areas in order to assess the causal influence of one brain site on another. Our hypothesis again was that the flow of information would originate from early sensory areas, and propagate to more anterior regions, utilizing the vOT as a central node, and that there would be feedback within this network, given the reciprocal connections existing between these brain areas.

## Materials and Methods

### Ethics Statement

The experiment was approved by the Behavioural Research Ethics Board of the University of British Columbia and all participants provided written consent.

### Participants

Fifteen right-handed volunteers (10 male) attending the University of British Columbia, age 18–35 years (mean = 22, sd = 4.26), were paid to participate. All participants indicated English as their first and primary language, and no histories of neurological, learning, or reading disorders or dysfunctions were reported during a prescreening interview. All participants had normal or corrected-to-normal vision, and indicated hand preference with the Edinburgh Handedness Inventory [22].

### Experimental Procedures

**Figure 1:**
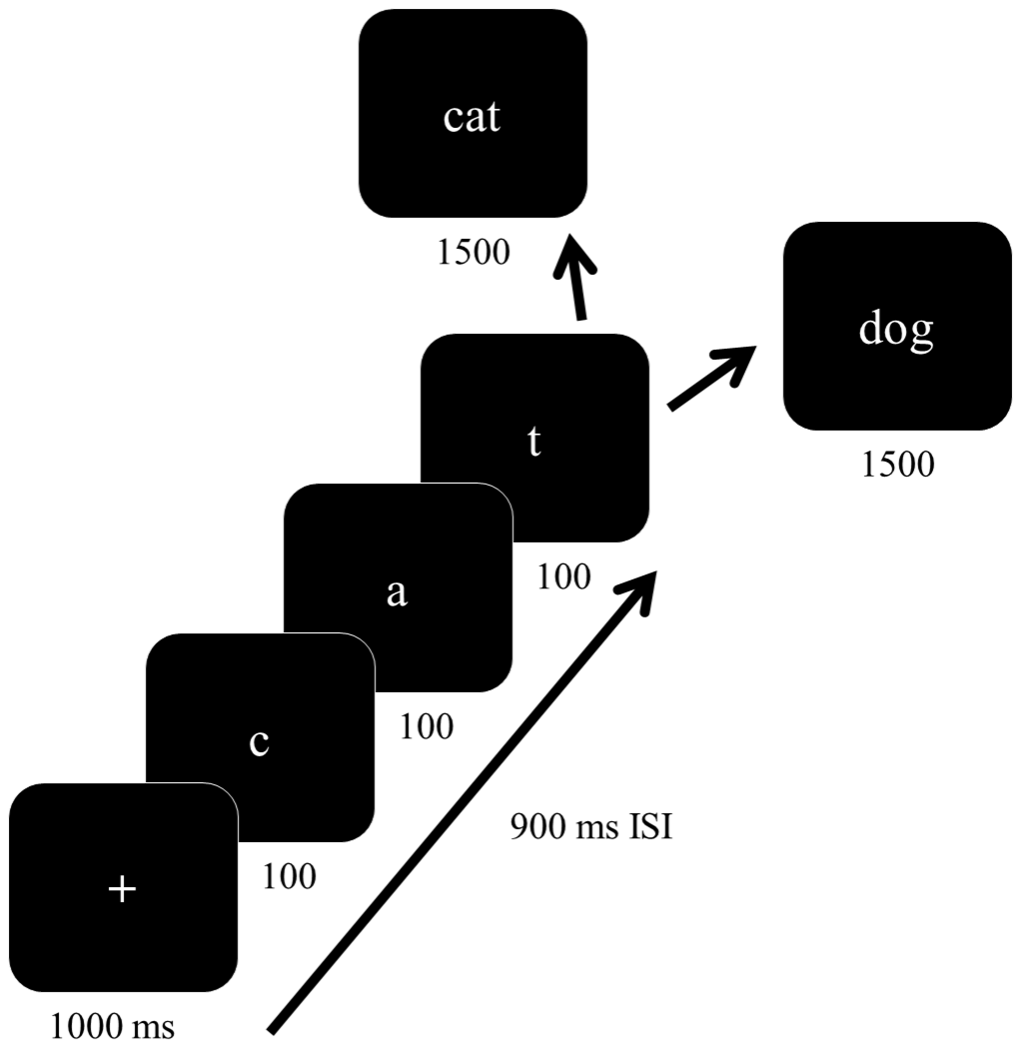
A schematic of the stimulus presentation and task. After a three letter sequence is presented, volunteers must respond as quickly as possible with regard to whether or not the word that appeared matched the word spelled by the letter sequence that came before it.

Participants were instructed to observe a sequence of three individual letters, followed by a three-letter word. The participants’ task was to respond on a keyboard as to whether or not the word that appeared matched the word formed by the letter sequence that came before it (Fig. 1). All letter sequences and words were three letters long, and were sourced from a pool of 456 possible words. Each trial had a 50% chance to be either a match or non-match trial. Letters and words were presented in 65-point Times New Roman font on a high-resolution CRT approximately 65 cm from participants’ eyes, and the font color was white on a black background.

In order to deter participants from simply comparing the forms of the shapes on the screen instead of actually reading the words, we warned participants to read the entire word that appeared after the sequence, as some letters would occasionally appear in both the sequence and word, but the two would still be a non-match (e.g. C-A-R and CAN). Furthermore, as shown in Section 3.2, brain sites preferentially responsive to reading and semantics were found to be generators of neural activity in this task, indicating that the words were read beyond basic form processing. Finally, whether or not participants intended to read the word, robust phenomena such as Stroop interference [23] further lend credence to the assumption that words are read automatically in such tasks.

Participants completed a practice session of ten trials prior to the experimental task and were offered extra practice trials if needed (no one needed extra practice). Trials began with a fixation cross for 1000 ms, followed by each of three letters being presented for 100 ms, with a 900 ms inter-stimulus interval. Finally a three-letter word appeared, at which point the participant was required to respond. The word remained on the screen for 1500 ms. Trials were separated by a 900 ms inter-trial interval, and a 30 s break was given after each 50-trial block. Each participant completed nine approximately 50-trial blocks, comprising a total of 228 each of match and non-match trials. Only trials on which a correct response was made were analyzed further.

### EEG Recordings and Analyses

EEGs were recorded from 60 passive electrodes in a standard electrode cap (Electro-cap, Inc., Eaton, OH, USA) at equidistant locations based on the International 10–10 System, referenced to the mastoids with the ground at AFz. EEG signals were amplified and sampled at 500 Hz through an analog passband of 0.01–100 Hz (SA Instrumentation, San Diego, CA, USA). Eye muscle activity was recorded by electro-oculogram (EOG) from four periocular electrodes. All electrode impedances were below 10 kΩ (input impedance of the amplifier was >2 gΩ).

Prior to analysis, all signals were re-referenced to an average reference, resampled to 250 Hz, and digitally filtered from 1–50 Hz using EEGLAB software [24], an open source MATLAB toolkit (MathWorks, Natick, USA), and custom scripts. First, the continuous data were epoched into 6 s bins time-locked to the presentation of the word. The epochs captured 4 s before and 2 s after word presentation. Epoching was performed in order to contain only the relevant processing associated with the task (letter and word processing), and not the inter-trial activity, or the activity occurring during breaks between blocks, so as not to introduce extraneous noise into the independent component analysis.

### ICA and Dipole Fitting (Source Localization)

Independent component analysis (ICA) is a method of blind source separation that takes into account the information from all EEG channels, and produces an equal number of statistically maximally-independent signals, termed independent components (ICs), free of the volume conduction that characterizes the raw scalp EEG [25], [26], [27]. This is accomplished by iteratively solving a neural network for an IC x channel “unmixing” matrix, *U*, according to *I = UX*, where *X* is the electrode channel x time data matrix and *I* is the IC x time matrix of independent component activations. The ICs represent non-Gaussian neural sources that are maximally independent of each other in the sense of sharing minimal mutual information. They represent new ‘virtual channels,’ information sources that, when re-mixed according to the inverse of the unmixing matrix, reproduce the original scalp data. We computed the ICA using EEGLAB’s *runica* function, which implements extended infomax ICA [25], [26].

We localized the brain sources of the ICs using the *dipfit* algorithm in EEGLAB. Electrode locations were co-registered to the Montreal Neurological Institute (MNI) average brain, allowing for Talairach coordinates to be produced for the IC dipoles. ICs with dipoles sourced outside of Talairach brain space were rejected as artifacts, and only ICs fit by single dipoles with less than 15% residual variance were considered for further analysis. Importantly, subsequent spectral power and connectivity analyses were performed *on the IC activations themselves*, and *not* on dipole activations. The dipole localizations served as an important tool for interpretation of the IC activity, but they, and the brain model they utilized, had no bearing on any other analyses.

We conducted a cluster analysis of all retained ICs, based on their associated dipoles’ locations in Talairach brain space, in order to identify common brain sources across participants. Twenty-five clusters composed from a total of 442 ICs (ranging from 29 to 47 ICs contributed by each participant) were created using EEGLAB’s *k-*means clustering algorithm, which minimizes intra-cluster distances while maximizing the inter-cluster distances, based on each IC’s dipole location in Talairach space. Large values of *k* relative to the number of ICs yield many small, highly-localized, clusters with only a few ICs per cluster, whereas small values of *k* yield a few large, diffuse, non-localized, clusters with many ICs per cluster. Given the goal of finding task-related ICs whose dipole localizations cluster tightly in a small brain region and that contain ICs contributed by the majority of participants, we investigated a range of *k*-values from 15 to 30. Although our results are based on a clustering with *k* = 25, other *k*-values in this range yielded virtually the same ROI-related clusters. We then selected the ICs comprising only those six of the 25 clusters that were localized to reading-related ROIs for further analyses. Although several of the unselected clusters are of some interest, their ICs were not analyzed further in the interest of our focus on reading-related activity and of minimizing statistical error.

The six selected clusters contained multiple ICs from several participants. Once these clusters were selected, they were pruned to contain only the most representative IC from each participant so as to weight each participant’s contributing ICs (in this case one IC per participant) equally. Pruning generally consisted of choosing the IC in closest proximity to the centroid and with the lowest residual variance (best fit to a single dipole), although in a few ambiguous cases we were forced to examine scalp maps and ERPs, selecting the cleanest one, to determine inclusion of one IC over another. At this point the power spectra of all included ICs were examined to ensure that they were characteristic of brain sources (roughly 1/*f*) and did not represent stereotypical artifacts such as eye movements or muscle twitches [28]. Because of the pruning process, and the similarity of the cluster results over a range of *k* values, the final clusters of ICs chosen for further analysis would have been the same regardless of initial clustering over the range of *k*-values that yielded relatively tight, representative dipole clusters.

We performed wavelet analyses on the included ICs in order to decompose the broadband signals into their component frequencies. Specifically, a Morlet wavelet analysis on each IC time series yielded wavelet coefficients of the sinusoidal oscillations between 1 and 50 Hz, from which amplitude (square root of power) and phase at each time-frequency point were calculated to be used in computing event-related spectral perturbation and phase synchrony analyses, respectively.

### Event Related Spectral Perturbation

Event-related spectral perturbations (ERSPs) were computed from the wavelet coefficients to generate a time-frequency decomposition on the retained ICs of the clusters of interest [29]. ERSPs allow us to observe the moment-to-moment fluctuations in oscillatory power, between 1–50 Hz relative to a baseline (−250 to −50 ms before the presentation of the first letter of a trial), that characterize an IC cluster. All ERSPs were masked at the single-subject level with a significance threshold of *p* = 0.01 by EEGLAB’s permutation test as there is no established error theory for such data. The EEGLAB test is a non-parametric test in which 100 (in this case) random shufflings of the ERSP data values are done to create a surrogate distribution of ERSP values for each time-frequency point. This technique preserved the actual values of ERSPs but scrambled them across trials and time-frequency points, so that they represented random assignments of those values to time-frequency points and trials, to create the surrogate distributions. If the real ERSP value for a time frequency point lay outside this distribution it was considered to be significantly different from zero at *p* = 0.01. Group significance was computed for each time-frequency point by a binomial test at a significance threshold of *p = *0.0001 [30]. In this test, the probability of a “success” (i.e. an individual permutation test significant by chance) was set at 0.01 (the significance threshold for the individual permutation tests) and the probability of a failure at 0.99 (1 minus the probability of a success). We considered the average ERSP to be significant only for time-frequency points where the probability of having the observed number or greater of individual tests significant at *p = *0.01 by chance (a “success”) was 0.0001 or less. Finally, in order to control family-wise error, only large (>100 time-frequency points) clusters of contiguous time-frequency points for which the group data were significant were considered to be meaningful [30]. Such large contiguous clusters have a vanishingly small probability of occurring by chance given the low probabilities of independent individual time-frequency points being significant in the group test. Nonetheless, contiguous time-frequency points are not independent. Therefore, we assumed that for any given contiguous large cluster, the probability of the entire cluster occurring by chance was that of any of the individual points in the cluster, in this case *p* = 0.0001. For the 6 ROIs, and considering we were only interested in whether a large cluster occurred in theta, alpha or gamma bands and in any of eight 100-ms time intervals, there would be 6×3×8 = 144 such tests. Thus the Bonferroni family-wise probability of Type I error for ERSP tests was 144×0.0001 or 0.0144.

### Phase Synchrony

Phase synchrony analyses were conducted in order to assess inter-regional functional connectivity, or the degree to which two brain areas are sharing information. This was done by computing the phase-locking values (PLVs) between pairs of ICs localized to specific brain regions. PLV’s were computed using the following formula (Delorme and Makeig, 2004):
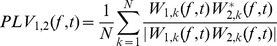
where *W_i,k_*(*f,t*) are the wavelet coefficients for each time point, *t*, and frequency, *f*, for each IC, *i*, and *k* = 1 to *N* is the index of epochs. The PLVs produced by these computations indicate the degree of constancy of the phase differences between signals at a specific oscillatory frequency across trials. PLVs range from 0 to 1, where 0 indicates the absence of any phase locking, and 1 indicates perfect phase locking, such that the phase difference between two ICs at a given time point remains constant across all trials. Only stochastic phase locking, with 0<PLV <1, is expected from any time series of brain activity because of neural noise [31].

PLVs were computed on each pair of component clusters by identifying the ICs from participants that were common to both clusters. Permutation statistics [32] were used to construct a surrogate distribution (N = 1000 permutations) for each time-frequency point with which to compare the obtained PLVs. Only PLVs significant at *p* = 0.001, that is, the obtained PLV lay outside the surrogate distribution, were considered to be meaningful. This analysis was performed for each participant in each component cluster separately. Group significance was determined with a binomial test as for the ERSPs, but with probability of success set at 0.001 (probability of a individual permutation test being significant by chance) and with a threshold probability of *p = *0.000001 for the probability of having the observed number of individual tests significant by chance. As for ERSPs, only large clusters of contiguous significant time-frequency points were considered to be meaningful. We required that at least 24 ms (6 successive time points) of a 50 ms interval at any frequency within the relevant frequency band be significant by the group test. There are sixteen 50-ms intervals in the 800 ms epoch, 51 frequencies in the two frequency bands (theta and gamma), and 15 pairs of ROIs, for a total of 16×51×15 = 12,240 tests. Again assuming that the probability of such a cluster occurring by chance is that of any of the individual time-frequency points because of non-independence of contiguous points, the Bonferroni family-wise probability of Type I error would be 12240×0.000001 = 0.012240.

Issues of spurious PLVs or the entrainment of oscillations by volume conduction are avoided here by comparing maximally independent signals obtained through ICA. These concerns are further assuaged by the lack of near-perfect PLVs (>0.8), which would be a marker for the entrainment of signals; instead PLVs generally were smaller than 0.3. Moreover, the phase lags of the significant PLVs were always substantially different from zero (not clustered near or around zero phase lag), indicating that volume conduction, which can cause zero-phase-lag synchronization, could not have been responsible for the significant PLVs.

### Transfer Entropy

Whereas measures of functional connectivity show which brain areas are engaged and sharing information, these measures do not indicate the causal flow of the information. That is, a measure such as phase synchrony does not indicate which site is *sending* the information, and which site is *receiving* the information, or if a bi-directional relationship exists. In order to understand such relationships, effective (causal) connectivity analyses must be employed. One such analysis that is commonly used is Granger causality, or variants thereof. A disadvantage of this method, however, is that it assumes, *a priori*, a linear model of the interaction between neural sources [33]. A linear model may become problematic when trying to determine causal relations in a highly non-linear system such as the brain. For this reason, we adopted a recent technique called transfer entropy, as it can determine causal interactions, including especially non-linear ones, without needing to specify or fit a model [34], [35]. Transfer entropy from time series *J* to time series *I* is defined (Schreiber, 2000) as the (asymmetric) Kullback-Liebler entropy between two time series at a specified, non-zero, lag (*k*–*l*):
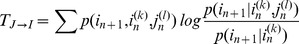



Transfer entropy measures the extent to which the transition probabilities (dynamics) between states within one time series (say *J*) are *not* independent of the past states of another time series (say *I*). It is larger the greater the influence of the state of *I* on the transition probabilities of *J*. We computed narrow-band transfer entropy (NBTE) on FIR-filtered (using EEGLAB’s firls() function) EEG amplitudes for theta (3–7 Hz) and gamma (35–45 Hz) frequency bands between all pairs of IC activations from the same participant(s) located in vOT and in the other clusters of interest. NBTE computes the information transfer at specific frequency bands rather than that of the broadband signal; this approach allows for examination of cognitively relevant aspects of various frequency bands (e.g. theta and gamma). We used a MATLAB toolbox called TIM provided by German Gómez Herrero and Kalle Rutanen (http://www.cs.tut.fi/~timhome/tim/tim.htm). TIM computes composite NBTE at each time point based on multiple replicates of each time series [36]. In the present case, the multiple replicates were the roughly 438 epochs (correct trials per participant) of theta-band (3–7 Hz) activations of each pair of ICs. We examined NBTE at lags between 30–50 ms for each 50 ms time bin of the epoch. An individual participant’s NBTE was considered to be significant for a given 50 ms time bin if a surrogate analysis (provided by the TIM toolbox) was significant at *p_I_* = 0.05 for at least 24 ms (6 successive time points) for a given lag between 30 ms and 50 ms. Because of the slight differences in lags and specific time points (to be expected when testing a diverse group of participants) at which the individually significant results occurred, it was not possible to simply average NBTE across individuals to show a group result. Therefore we had to resort to a binning procedure for the group results. All significant directed transfers are based on group-level significance threshold of *p_G_* = 0.01 (probability of the observed number, or greater, of the individual tests being significant by chance) determined using a binomial probability test as for ERSPs and PLVs. Here, however, the probability of a success in the binomial was set at 0.05, which was the significance threshold for the individual tests. We combined pairs of 50-ms bins into 100 ms intervals; we only considered a group result to be meaningful if it was significant for *each* of the 50-ms bins in the interval. We considered time periods when either only one or no 50 ms bin reached significance by our group criterion during that period to be non-significant, although in most cases several individual participants did display significant NBTE and often the group result reached significance in one or the other but not both 50-ms bins. As the probability of a significant group result in a given 50-ms bin by chance was *p = *0.01, and assuming independence of successive 50-ms bins, the joint probability of two bins being significant in a single interval is 0.01^2^ = 0.0001. There are 15 pairs of ROIs and eight 100 ms intervals and two directions for a total of 15×8×2 = 240 tests. The Bonferroni family-wise probability of a Type I error in these tests is thus 240×0.0001 = 0.0240.

## Results

### Behavioural Results

Participants completed the word reading task with a mean accuracy of 96.0%. Of the correct trials, participants showed no significant difference in reaction times between the match (*M = *532 ms, *SD = *140) and non-match (*M = *546 ms, *SD = *161) trials, *t*(14) =  −1.04, *p = *0.32. Correct match and non-match trials were thus collapsed for all analyses of brain activity; this amounted to approximately 438 trials per subject. Here we focus on brain responses to the whole word presentation on each trial, although responses to the preceding letters will sometimes be relevant.

### EEG Localization Converges with Neuroimaging Data

**Figure 2:**
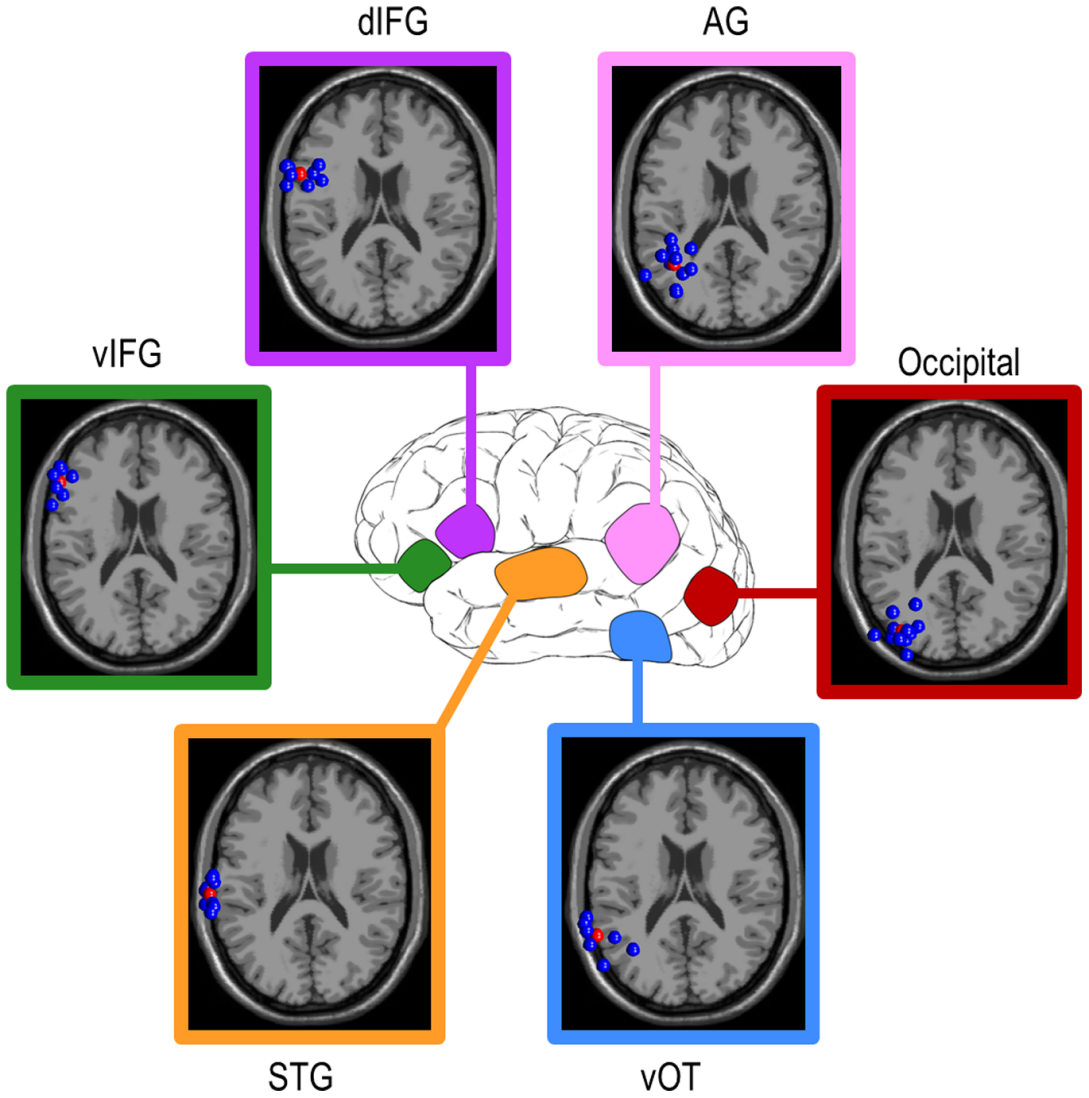
Selected dipole clusters in three-dimensional Talairach space. ICA and dipole fitting yielded multiple regions related to reading. Within each horizontal slice, individual participants’ ICs are represented by blue dots. Red dots are the cluster centroids; for each cluster the average residual variance of included ICs is less than 6.5% (see Table 1). The indicated z-coordinates are those of the cluster centroids (red dots). vOT ventral occipito-temporal cortex; AG angular gyrus; STG superior temporal gyrus; IFG inferior frontal gyrus; d dorsal; v ventral.

**Figure 3:**
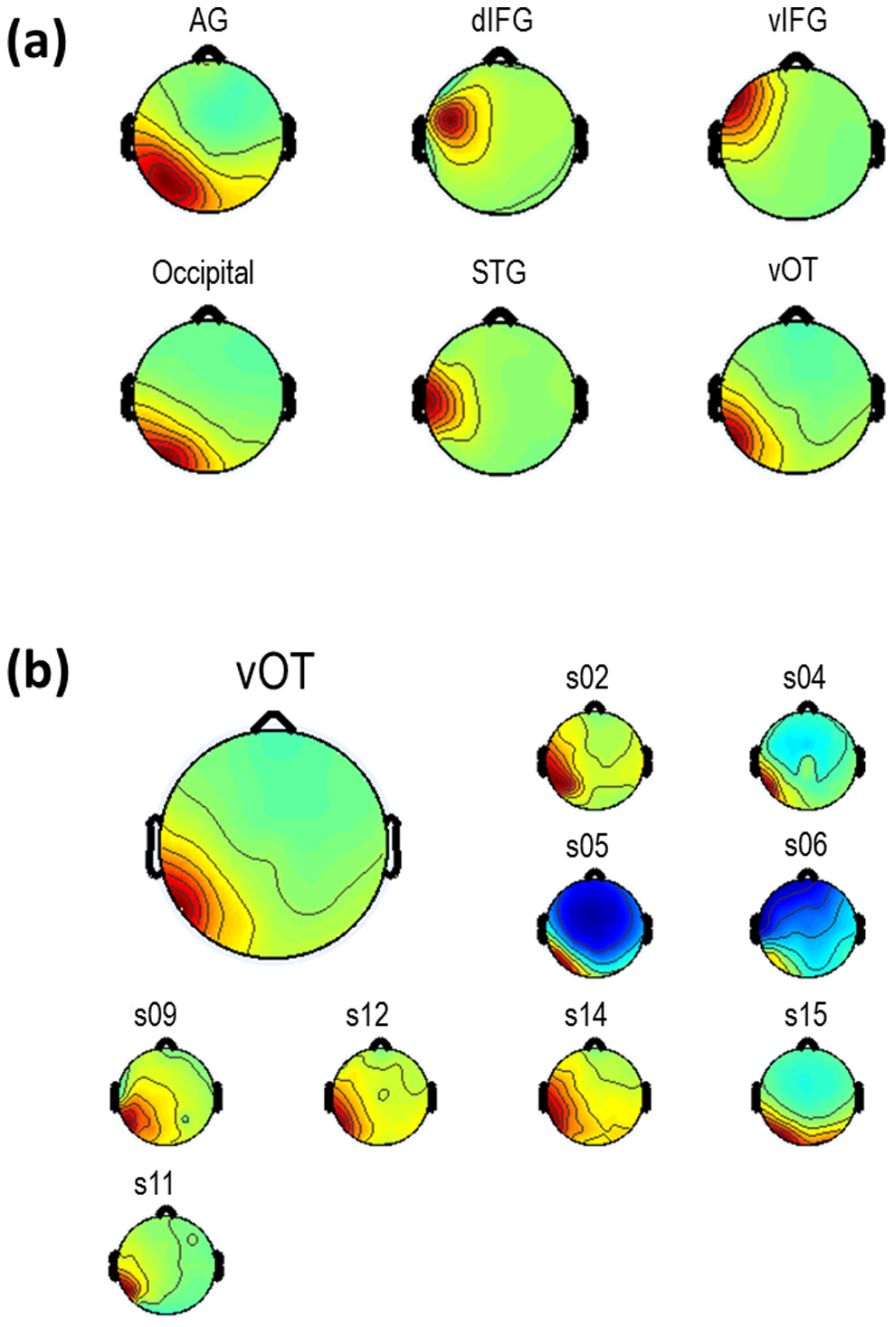
Scalp topographic maps of ICs. (a) Topographic maps of group average ICs for the ROIs shown in Figure 2. (b) Topographic maps of the individual participants’ ICs for the vOT illustrate their resemblance to the group average IC. Results are similar for other ROIs. vOT ventral occipito-temporal cortex; AG angular gyrus; STG superior temporal gyrus; IFG inferior frontal gyrus; d dorsal; v ventral.

Figures 2 and 3 display the results of the ICA and dipole fitting followed by the cluster analysis used to group valid ICs in order to identify active brain regions that were common across participants. Importantly, scalp maps of valid IC weights clearly depicted single dipole sources, as expected from and typical with ICA (Fig. 3a), affording us the interpretation that these locations represent compact cortical generators [26]. The single dipole topographic maps are apparent at the single participant level as well, and within a cluster the maps from single participants’ ICs were highly similar to that for the centroid (Fig. 3b for an example for the vOT). The ERP localized to the vOT dipole cluster showed a negative peak at ∼170 ms (N170; Fig. S1), as expected for word processing [12]. Moreover, the IC clusters were localized to ROIs derived from previous fMRI and PET studies with high accuracy (Table 1). Although minor discrepancies do exist between the two sets of coordinates, these differences likely arise from the fact that the imaging meta-analysis examined only the maximally active voxel in a given activated region, rather than the spatially central voxel. These results strongly support the conclusion that we have identified and recorded the EEG activations of the primary brain regions associated with word reading.

**Table 1:** Talairach coordinates (±SD) of IC cluster centroids and their distance from comparable neuroimaging results.

	vOT	AG	vIFG	dIFG	STG
Brodmann Area	21/37	39/22	47	45/44/9	22/42
%VAF	93.6	95.6	95.3	95.5	95.0
No. of participants in cluster	9/15	12/15	7/15	9/15	11/15
IC cluster centroidcoordinates (SD)	−62(12), −50 (11),−5(8)	−39(9), −52(12),22(8)	−57(5), 25(10),−7(10)	−57(11), 10(6),23(11)	−71(3), −22(9),8(10)
Jobard et al. (2003)centroid (SD)	−44(4), −58(5),−15(6)	−60(4), −41(6),25(6)	−44(4), 23(6),17(3)	−50(5), 10(5),4(8)	−53(6), −13(7),0(4)
Euclidian distance from ICcluster centroid	22.09	13.60	27.37	20.25	12.00
Vigneau et al. (2006)centroid (SD*)	–	−45, −68,26 (14.1)	−43, 20,4 (16.0)	−48, 2,26 (9.6)	−60, −27,9 (8.4)
Euclidian distance from ICcluster centroid	–	17.55	18.5	12.41	12.12

**VAF** average scalp potential variance accounted for by dipoles in cluster; **OT** occipito-temporal; **AG** angular gyrus; **STG** superior temporal gyrus; **IFG** inferior frontal gyrus; **v** ventral; **d** dorsal. Regional locations based on centroid mean; although IC clusters are quite tight, occasional ICs within a cluster fall slightly outside the listed region. *Vigneau et al. computed SD as the square root of the mean of squared Euclidian distances to the cluster centroid.

### ERPs and Event-related Spectral Perturbations

**Figure 4:**
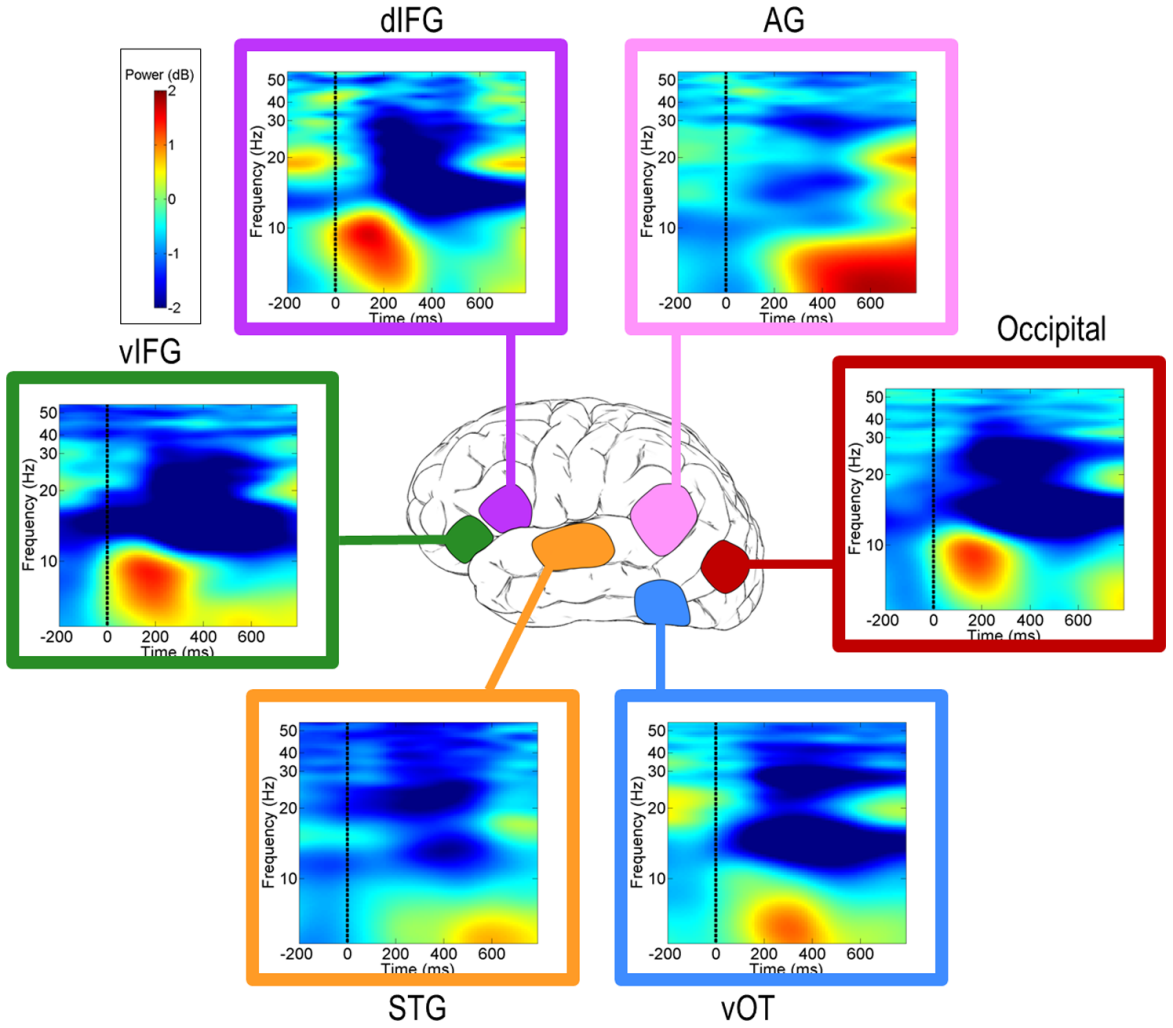
Average ERSPs for the IC clusters. Power in dB with respect to a baseline from −250 to −50 before presentation of the first letter. All ROIs show a prominent burst in theta (3–7 Hz) power, the earliest activity occurring immediately after word presentation (<50 ms), and the latest occurring ∼600 ms after the onset of the word. vOT ventral occipito-temporal cortex; AG angular gyrus; STG superior temporal gyrus; IFG inferior frontal gyrus; d dorsal; v ventral.

Although the purpose of this study was to characterize connectivity dynamics between brain regions, we first had to verify that the localized activity was task-relevant. This was determined using ERPs (particularly for the vOT, Fig. S1) and event-related spectral perturbations (ERSPs). We observed theta-band (3–7 Hz) ERSP increases in all ROIs beginning shortly after the presentation of each letter (not shown) and of the word (Fig. 4). Increased gamma-band (30–50 Hz) activity also occurred around the presentation of each letter (not shown) and word, particularly in dIFG and AG (although not readily apparent in Figure 4, peak ERSP power at word onset was 0.15 dB and 0.40 dB, respectively, significant by permutation test). Alpha-band (8–14 Hz) suppression was observed after each letter and more strikingly after the word presentation (Fig. 4). Finally, there was a burst of increased beta-band (15–30 Hz) activity shortly after the time of the response, which occurred at approximately 540 ms post-word (Fig. 4).

### Functional Connectivity over Time in the Reading Network

**Figure 5:**
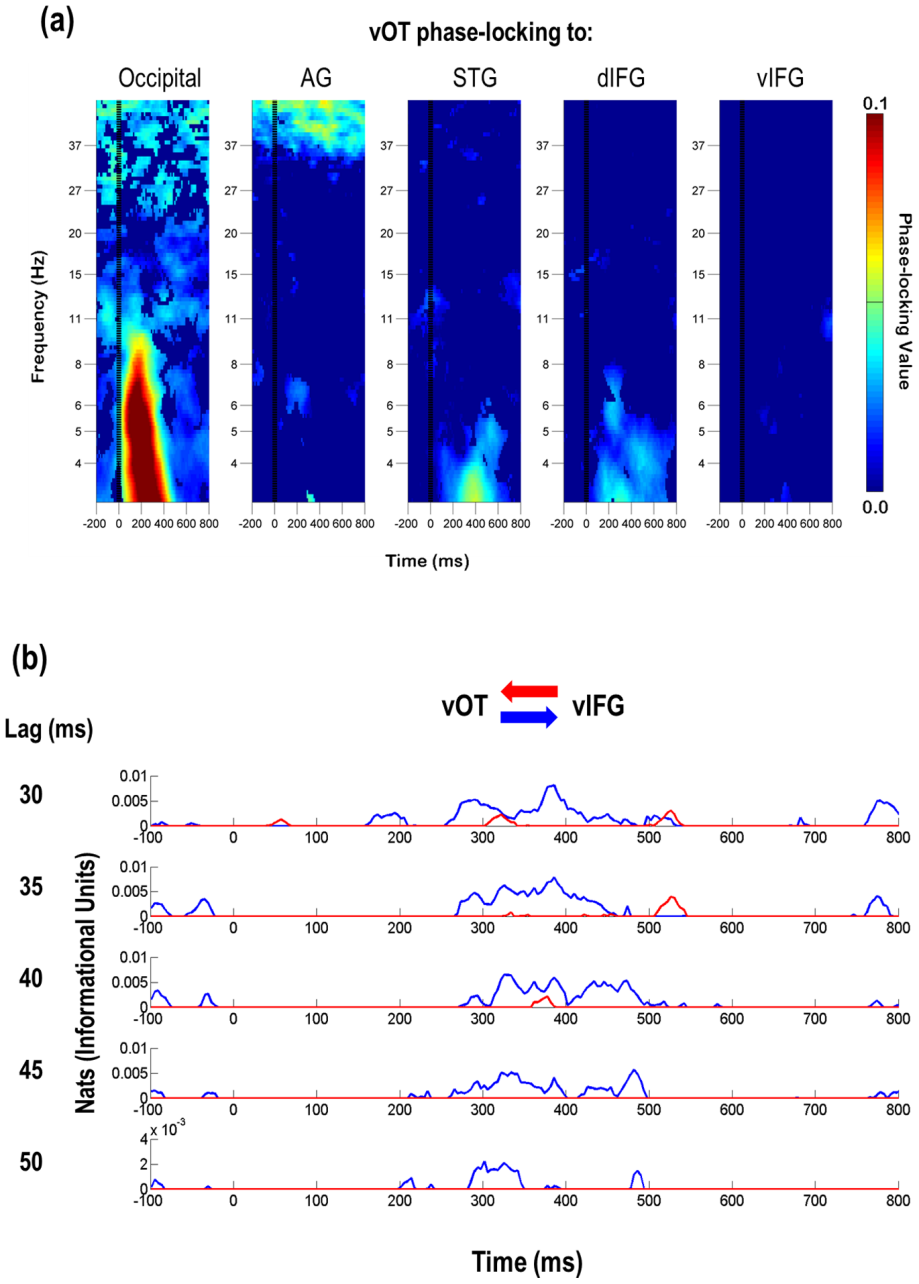
Time-frequency plots of PLVs and outputs from narrow-band transfer entropy (NBTE) analysis. (**a**) vOT shows distinct patterns of functional connectivity among the regions in the reading network. PLVs are masked for significance at *p* = 0.001 by permutation test for individual participants and then again at *p* = 0.000001 for the group (see Methods). (**b**) Example of theta-band NBTE (30–50 ms lag) between vOT and vIFG from a single subject. The plot depicts the flow of information between areas, where the blue line represents the transfer of information *from* vOT *to* vIFG, and the red line represents information flow from vIFG to vOT. In this case, across all lags, vOT sends information from ∼250–470 ms, followed by vIFG sending information back at ∼500–550 ms, indicating an initial feed-forward response, followed by feedback to the original source. Also shown here are instances in which both NBTE patterns overlap, signifying a bi-directional relationship (e.g., ∼300–400 ms). NBTE results for individual participants were masked for significance at *p* = 0.05 by permutation test (see Methods). vOT ventral occipito-temporal cortex; AG angular gyrus; STG superior temporal gyrus; IFG inferior frontal gyrus; d dorsal; v ventral.

**Figure 6:**
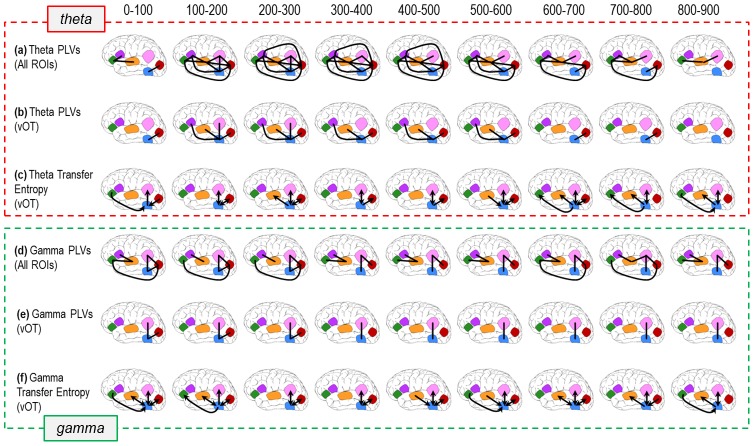
The temporal evolution of functional and effective connectivity after viewing a word. All connectivity analyses were computed over the span of the entire 6(**a**) Theta-band PLVs (significant functional connectivity, black lines – see Methods) among all ROIs, evolving over the course of viewing a word. (**b**) Theta-band PLVs relative to vOT. These depictions of connectivity are derived from (a), isolated to bring focus to vOT’s widespread involvement in the reading network. (**c**) Theta-band NBTE (causal connectivity, black arrows mean that NBTE was significantly different from 0 in both 50-ms bins in 100-ms interval – see Methods) relative to vOT, showing a comparable pattern of network engagement to PLVs (b). This also highlights some of the uni- and bi-directional relationships that exist in the time-course of word reading. (**d**) Gamma PLVs show similarities to their theta counterparts, albeit with sparser connectivity overall. This is further highlighted when vOT gamma interactions are isolated in (**e**), illustrating their confinement to posterior cortices. (**f**) Gamma-band NBTE (black arrows) relative to vOT, showing a comparable, and perhaps even more robust, pattern of network engagement relative to PLVs (e).

Figure 5a depicts time-frequency plots of PLVs between area vOT and each other ROI. These data (and those from all other ROI pairs – see Fig. S2) were used to create the functional connectivity maps in Figure 6. Figure 6 displays the summary mappings of significant PLVs in the theta- (Fig. 6a, 6b) and gamma- (Fig. 6d, 6e) bands. Inspection of vOT theta-band PLVs over time shows widespread connectivity across the entire left hemisphere (Figs. 5a, 6b). Within the first 100 ms after word presentation occipital and vOT sites show strong engagement that persists until 800 ms. From 100–600 ms vOT is connected to dIFG and STG and from 100–300 ms to AG as well. In addition, vIFG is connected to dIFG and STG from directly after word presentation until 800 ms (Fig. 6a). Indeed, from about 100 ms on robust increases in theta-band phase-locking are observed among most ROIs until finally tapering off at ∼800 ms (Fig. 6a).

Gamma-band synchronization also was distributed widely throughout the left hemisphere. It followed a similar time course to that of theta-band synchronization, with some differences in the particular regions involved (Figs. 5a, 6d, 6e). From very shortly after word onset to 300 ms vOT shows phase-locking with occipital and AG regions, at which point vOT remains solely engaged with AG until ∼800 ms. Occipital cortex also shows increased PLV with vIFG and AG until 300 ms post-word, and then again at 600–800 ms post-word, and STG also shows increased PLV with dIFG. Because synchronization in the gamma band was weaker and more intermittent between vOT and most other regions (except for AG), however, we decided to focus on the theta band for analyses of effective connectivity.

### Effective (Causal) Connectivity

Figure 6 displays the summary results of the NBTE analysis for both theta (Fig. 6c) and gamma (Fig. 6f) frequency bands. We computed theta- and gamma-band NBTE between ICs localized to the vOT and those localized to other relevant regions in the left hemisphere, including early visual cortex, left STG, vIFG, and dIFG. We did not compute NBTE for all pairs of ROIs in the interest of controlling false discoveries. An example of a typical theta-band result for a single subject and a single pair of regions is shown in Fig. 5b; Fig. S3 contains additional examples of single subject NBTE results for both theta and gamma bands (too many to display in full for all ICs).

The vOT is both a source and a receiver of information transfer during word reading in both theta and gamma frequency bands, showcasing striking similarities between theta and gamma in addition to some band-specific trends. One prominent similarity between theta and gamma is the NBTE among early visual cortex, vOT, and AG beginning immediately after stimulus onset and sustaining through the epoch until finally tapering off at ∼700 ms. This posterior sub-network shows robust bidirectional information transfer between vOT and critical visual and language processing centers during word reading.

Furthermore, within the first 100 ms, vIFG sends top-down signals to vOT in both frequency bands. However, a return signal from vOT to vIFG is only seen in the gamma band from 100 ms to 200 ms. With regard to STG engagement with vOT, theta and gamma are similar later in the epoch (500 ms to 900 ms), showing almost a complete overlap in directed information transfer in this pair of regions, save for a moment of feedback from STG to vOT from 600 ms to 700 ms in the gamma band.

Along with similarities, differences between the theta- and gamma-band NBTEs also exist. From word onset to 200 ms post-onset, bidirectional gamma NBTE is seen between vOT and STG, followed by feedback from STG to vOT from 400 ms to 500 ms. Theta-band activity yielded considerably less early NBTE, only showing information flow from vOT to STG from 200 ms to 300 ms. Later in the epoch, theta-band NBTE is seen flowing from vOT to vIFG from 600 ms to 800 ms.

Finally, a number of individual participants showed continuing significant theta-band vOT-dIFG interactions but the group results did not reach significance. Also notice in Figure 6c and 6f the interactions among vOT, vIFG, AG, and STG between 600 ms and 900 ms, some time after a response had been made to the stimulus word (at about 540 ms).

## Discussion

We studied the cortical network dynamics underlying word reading. In particular, we characterized the fast connectivity dynamics of the associated network processes. As expected, ICA and dipole fitting revealed neural electrical sources that converged remarkably well with existing fMRI (BOLD) and PET neuroimaging evidence [5], [37]. This is the first time to our knowledge that ICA and single dipole fitting have been used in this way to localize EEG activations in word reading. We cannot say with complete certainty that the localized ROIs from our data do, in fact, represent their corresponding neuroimaging counterparts, but there is much evidence to support this idea. In addition, our localizations also correspond well with another similar EEG study [38], although the techniques used for dipole localization were radically different. Additionally, semantic processing areas such as vIFG and the angular gyrus (AG) were identified. With regard to grapho-phonological processing, the STG and dIFG area were established as neural sources. With these reading-related brain regions verified, we then conducted connectivity analyses. In particular, we examined the dynamics of theta- and gamma-band oscillations, which have been implicated in basic perceptual, attention, and learning processes [39], [40].

### Feed-forward and Feedback Processes in Area vOT

Electrophysiological studies have thus far focused on patterns of activation during reading, capitalizing on principles of hierarchical visual organization. Functional representations of this hierarchy are congruent with commensurate anatomical hierarchies, and are exemplified in activation studies as a posterior-to-anterior sweep. Not explicitly seen in these activation maps, however, are indicators of feedback.

Recently, Price and Devlin [41] explicitly acknowledged the mechanism of moment-to-moment reciprocal relations between brain regions as being crucial to reading. This so-called Interactive Account operates on the premise that perception involves evaluation and resolution of automatic top-down predictions and bottom-up sensory information. Specifically, it posits that in reading, this process is resolved in area vOT, holding it as the critical interface between bottom-up visual information and top-down semantic and phonological representations. As these reciprocal connections are strengthened (through learning), the higher-level language areas can deliver predictions to vOT in order to facilitate the bottom-up visual processing, thus making the reading process more efficient. This framework is consistent with accounts of re-entrant processing in the brain, whereby perceptual representations are sustained by reverberatory loops in cortical processing so as to make the sustained information available to be used by other networks [42]. Herdman [16] speculated on this point further, indicating that oscillatory (i.e. gamma-band) activity in visual networks may represent a template-comparison stage, from which information is either sent to higher-level areas for further processing, or is maintained in a re-entrant loop for further evaluation.

In agreement with connectionist approaches [43], the framework further proposes that communication between vOT and language centers is not a serial process whereby the current active process, be it semantic or phonological processing, acts as the momentary relay in the propagation of the signal. Rather, signals from vOT operate in a cascade, continuously activating rudimentary semantic and phonological representations in language areas. Through reciprocal connections, these language areas then provide feedback to facilitate the evaluation of the orthographic pattern.

Of the various principles offered by this framework, here we focus on two: 1) Because of the reciprocal nature of the connections to and from vOT, much of the observed connectivity between vOT and other ROIs should show both feed-forward and feedback signals in the span of time involved in reading a word, and 2) The activation in vOT to word forms should activate–at least partially–semantic and phonological representations simultaneously in high-level language areas. In the present study, we document through connectivity measures the realization of both of these principles.

First, examination of theta-band NBTE (Fig. 6c) in the first 100 ms after word presentation shows bottom-up visual signals from occipital regions to vOT and from vOT to AG, as well as an automatic top-down predictive signal from vIFG to vOT. Immediately following this, however, feedback signals are seen from vOT to early visual sites, and from AG back to vOT, illustrating the emergence of a bidirectional relationship between vOT and other ROIs. Over the span of time involved in reading a word, similar feed-forward and feedback connectivity patterns were also seen between vOT and STG, as well as between vOT and vIFG. For example, in the gamma band, early (<200 ms) top-down signals from STG to vOT can be seen, potentially suggesting an instance of predictive coding in order to facilitate word processing (41). These results highlight the importance of backward connections in the propagation of the traditionally accepted feed-forward sweep of activation, although alternative study designs would be necessary to determine the precise impact of a given feedback signal.

Second, theta-band PLVs (Fig. 6b) showcase the cascade of engagement between vOT and language areas such as AG, STG and dIFG from ∼100–600 ms. Critically, these regions show early engagement with vOT simultaneously, all prior to their traditional windows of activation, usually occurring at ∼200–500 ms and all at disparate times [15], [44], [45]. Theta- and gamma-band NBTE results further corroborate this, showing early effective connectivity from vOT to areas AG and STG. These measures of connectivity provide evidence for the widespread, early simultaneous priming of language areas by vOT. The network dynamics presented here thus provide support for the Interactive Account of the reading process.

### Area vOT

Notably, the dipole fitting was able to identify a region in the fusiform gyrus, known as the visual word form area (VWFA) that consistently has been shown to respond especially strongly and selectively to orthographic stimuli [3]. Several previous studies of functional connectivity have also identified this region as an important component in a variety of letter and word reading tasks similar in some ways to ours. Our localization of this region is consistent with each of these studies, which include fMRI [46], EEG [38], and intracranial EEG [17] techniques as well as a variety of localization techniques. Among the ERPs shown in Fig. S1, area vOT shows a pronounced N170 component, a defining characteristic of ERP studies of orthographic processing [3].

As the pre-lexical processing center for reading, this area requires wide-reaching connections. It must be able to transmit orthographic information to higher level regions involved in language processing, namely for phonology and semantics. Our interpretation of area vOT as VWFA is supported by the fact that the theta-band phase-locking in the reading network was robust, showing functionally connectivity between vOT and four out of five other ROIs when reading a word. The positioning of this region offers it a broad reach across all language networks by way of white fiber tracts [7], [37], [47], [48], [49]. Our vOT cluster expresses this structural connectivity exceptionally well by engaging in theta- and gamma-band functional and effective connectivity at cognitively relevant times with occipital, AG, STG, and dIFG sites, each of which lie along the ILF and/or inferior fronto-occipital fasciculus.

### Angular Gyrus (AG)

Of the ROIs identified by two separate meta-analyses [5], [37], our AG dipole cluster was closest, by Euclidean distance, to AG in both studies (see Table 1). Multiple studies have implicated AG in word reading, often paired with Wernicke’s area, as with other language processing. Specifically, it is thought to be critical for semantic processing [37], although evidence of phonological processing exists [50].

Connectivity between AG and other sites in the reading network(s) remains severely under-explored. Pugh et al [50] reported functional connectivity between AG and occipital sites, as well as Wernicke’s area during a pseudo-word rhyming task. Our gamma-band synchrony and NBTE results (Fig. 6d, 6f) support this idea, showing what seems to be a posterior network, joining visual cortex and vOT and spanning the entire trial. Horwitz et al [51] showed similar results, reporting functional connectivity between AG and a variety of sites in the left hemisphere, including occipital and temporal cortices, fusiform gyrus, and inferior frontal cortex –all of which overlap with regions that we report here.

### STG

Our STG cluster is very close to the region labeled by Jobard et al [5] as a general STG reading region. Vigneau and colleagues [37] offer more specific localization, however, fitting this cluster into the planum temporale (PT), a highly lateralized subregion of the posterior STG in so-called association cortex, which has been shown to process a diverse range of complex sounds [52]. Until recently, PT was most associated with phonological processing. With regard to reading, the degree of PT lateralization in development has been shown to be related to reading ability. For example, Larsen et al [53] reported that adolescents with developmental dyslexia show a greater bilateral distribution of PT between hemispheres. The robust phonological impairments in developmental dyslexia imply critical role of PT in such processing.

The role of the superior temporal lobe in reading is not restricted to one linguistic function, however. In fact, Yvert et al [38] described moments of both distinct and overlapping semantic and phonological processing in this region during word reading. Thus, our STG cluster could be dynamically filling semantic and/or phonological processing roles over the course of viewing a word.

### IFG

Our cluster analysis yielded two distinct IFG clusters, one located more dorsally (dIFG), near the pars opercularis of IFG (poIFG) and precentral gyrus (PCG), and the second positioned more ventrally (vIFG), near pars triangularis of IFG (ptIFG) and lateral orbito-frontal cortex. Indeed, when compared to a meta-analysis of semantic and phonological centers of the brain [37], our dIFG cluster showed the closest proximity to the PCG and bordering on poIFG (see Table 1), while the vIFG cluster was closest to the ptIFG region. Beyond the anatomical separation, moreover, the literature supports distinct functional roles for the two IFG regions in reading and language. poIFG and PCG have generally been found to be critical for phonological processing [54], [55], and ptIFG has been associated with semantic processing [56], [57], [58].

In both PLV and NBTE results, early connectivity is seen between vIFG and visual areas immediately after word presentation (<100 ms). Early IFG activity is further indicated by dIFG and vIFG ERSPs, showing very early theta bursts (<100 ms) long before their characteristic appearance shown in studies that explicitly examine phonogical processing in reading [15]. Wheat et al [59] found a similar result when priming subjects with a masked pseudohomophone (e.g. masking ‘brein’ before ‘BRAIN’) in a visual word recognition task. They reported a very early (<200 ms) theta and gamma responses in the pars opercularis of IFG, as well as the precentral gyrus, suggesting an early phonological processing component to word reading. The present study, however, shows a similar effect without such priming. It is possible that the working memory component of the task, in which subjects are required to remember the three-letter sequence, accounts for this early frontal top-down engagement from IFG.

### Inter-regional Connectivity

Our hypotheses concerning the temporal evolution of theta-band phase synchrony were confirmed. Increases in theta-band PLVs with the vOT region first emerged in posterior visual cortex, and cascaded to higher-level areas over several hundred ms. Early synchronization at low frequencies reflects the patterns of activation seen in ERPs, and since low frequencies tend to dominate the ERP waveforms produced in the brain is at least partially attributable to evoked activity. The later theta-band synchronization, however, arises among induced oscillations.

Gamma-band synchronization, although showing a less well-defined spatio-temporal pattern of progression, still supported network connectivity during word reading, particularly between vOT, AG, and occipital cortex. This group of regions might form a subnetwork with a special task during reading (cf. [17]). Given the involvement of the AG in semantic processing, perhaps this is one particular circuit involved in accessing individual word meaning.

In addition to functional relationships established between brain areas using phase synchrony analysis, NBTE was used to determine the causal flow of information involved in those relationships. As expected, the vOT was intricately involved in information transfer in the theta band during word reading, reflecting and clarifying the theta-band phase synchrony results as well as elucidating the causal patterns in the gamma band activity. Notably, the NBTE results in both frequency bands showed both feed-forward and feedback flows of information from each region to the next, with sustained feedback signals being sent to lower areas during various time periods. This feedback is indicative of re-entrant processing following the initial feed-forward perceptual processing of the word stimulus [42].

In both theta and gamma NBTE, areas vOT, AG, and early visual cortex bidirectionally shared information for almost the entire epoch. These results are also reflected in the theta and gamma PLVs as well, showing almost the exact same pattern shortly (<300 ms) after word onset. This trio of ROIs may serve as a potentially critical posterior subnetwork for word reading responsible for initial processing prior to phonological access.

Unexpectedly, early (0–100 ms) top-down causal connectivity was observed between vOT and vIFG. This early integration of language network dynamics, however, is likely a property of the specific reading task we employed, as participants should come to expect and anticipate features of the word they are asked to process based on the sequence of letters they have just experienced. This anticipation and expectation seems to influence and constrain the processing at lower level areas in our paradigm.

One particularly interesting aspect of our NBTE results is that both the theta- and gamma-band network patterns of information transfer overlap greatly, with a few exceptions. This may seem odd, given that these frequency bands are spaced relatively far apart. However, these similarities adhere to existing frameworks of frequency interactions in the brain–most notably the idea that fundamental neural computations are processed at gamma frequencies, which are then distributed throughout networks via specific phase relationships with theta rhythms. In this way, gamma activity might be “carried” by theta waves [60]. However, this relationship also poses hurdles in the interpretation of some of our results. If gamma activity needs concurrent theta activity to propagate its information, it is somewhat odd that our NBTE results show gamma information transfer without said theta information transfer also occurring in the same time intervals. There are many potential explanations for the discrepancy. First, it may simply be the case that some NBTE links were not strong enough to exceed the statistical thresholds used in our analyses. Indeed, in some cases, certain links were trending in individual subjects, but such links failed to appear at the group level. In other cases, it is unclear whether the instances of information transfer were absent or if our statistical techniques were not sensitive enough to pick up all “true” moments of information transfer. Ultimately, we do not make a distinction as to whether solely theta or gamma activity is more responsible for enabling reading processing in the brain, but rather, the two frequency bands appear to be utilized in concert to maximize the information exchanged in the reading network.

### Limitations

Our study has some limitations that should be overcome in future research. First, using our task it is difficult to pinpoint single word processing without involving working memory (WM) components in the signal. That is, we cannot capture reading processes without also capturing WM processes. Our results need to be replicated using a more classical design (e.g. a lexical decision task) that does not rely on WM, in order to compare them to more established/known results. Nonetheless, there will always be some aspect of memory involved in any reading task, if only in lexical lookup. Moreover, because stimuli are presented sequentially, memories of previous stimuli will influence responses to current ones. Therefore, reading-specific process can only be isolated from a converging confluence of varied reading-related tasks.

If PLV and NBTE were both error-free, complete, descriptions of functional and effective connectivity, respectively, then we would expect to find functional connectivity everywhere we find effective connectivity, although not vice versa. But here the methods are being applied to noisy data, and neither has been shown to be error free – indeed statistical errors are inevitable. Moreover, given the noise in the data and differing notions of how to measure brain-regional functional and effective connectivity, neither is likely to be complete either. Thus, we do not find functional connectivity everywhere we find effective connectivity in these data – indeed our NBTE analysis seems to be more sensitive than the PLV analysis – although this may be partly because of somewhat different thresholds employed (more conservative for the PLV analysis because of the greater number of tests done there). Also, the two tests are very different mathematically and address the data in very different ways (oscillatory phase difference relationships versus information measures based on the filtered oscillatory signal). Thus, somewhat different results are expected based on the distinct theoretical assumptions underlying each technique (see Methods). Ultimately, the detailed relationships between these and other techniques used to reveal interactions between brain regions, such as dynamic causal modeling, remain to be explored further.

Another limitation is that we studied carefully only oscillatory activity in theta and gamma bands. It is possible that important connectivity during reading is occurring in the alpha and beta bands. Also, some of our sources (e.g., vIFG) could have been closer to the centroids indicated in the literature. Unfortunately, EEG caps simply don't cover all of the head, and we did not use individual subjects’ head models for our dipole fits, so localizations are somewhat imprecise. Nonetheless, we still localized ROIs in very close proximity to those identified in two meta-analyses, particularly to those from the phonological/semantic study [37], usually substantially less than 2 cm from our centroid to their most active voxel.

Finally, both our PLV and NBTE analyses were bivariate (done on individual pairs of ICs) and thus we could have some spurious PLV or NBTE results if a third IC were functionally or causally involved with both of the ICs tested. Given our conservative criteria we don’t believe this is a serious problem, but it could be alleviated by performing multivariate PLV and NBTE analyses.

## Conclusion

These results extend our knowledge of reading in the brain beyond the functional anatomy into the dynamics of information transfer within brain networks. The present findings demonstrate the moment-to-moment neural interactions during reading in ways that we have not seen previously, and provide useful strategies for identifying connectivity and timing alterations relating to language integration in individuals with reading challenges (dyslexia).

## Supporting Information

Figure S1 Dipole cluster ERPs. Area vOT shows a prominent N170 (negativity at ∼170 ms), a hallmark of categorical (and orthographic) processing. ERPs at other sites show similar, yet distinct ERP waveforms. vOT ventral occipito-temporal cortex; AG angular gyrus; STG superior temporal gyrus; IFG inferior frontal gyrus; d dorsal; v ventral.
(TIF)

Figure S2 Time-frequency plots of PLVs among ROI IC clusters except for vOT with the others, which is shown in Figure 5a. For all phase synchrony analyses, individual subject PLV significance was computed at *p* = 0.001 by permutation test, and group significance was determined with a binomial probability of *p* = 0.000001. vOT ventral occipito-temporal cortex; AG angular gyrus; STG superior temporal gyrus; IFG inferior frontal gyrus; d dorsal; v ventral.
(TIF)

Figure S3 Example single-subject results from theta (3–7 Hz) and gamma (35–45 Hz) narrow-band transfer entropy (NBTE) analysis. Blue lines represent significant (by surrogate at *p* = 0.05) NBTE between vOT and occipital, AG, and dIFG regions, red lines represent the same from each respective site to vOT. Instances in which both NBTE patterns overlap signify a bi-directional relationship. Each small graph represents a different lag from 30 to 50 ms.
(TIF)
