# Supplementary figures and images for: Fast Dynamics of Cortical Functional and Effective Connectivity during Word Reading
Source: PLoS One. 2014 Feb 14;9(2):e88940. doi: 10.1371/journal.pone.0088940 (PMC3925174; doi:10.1371/journal.pone.0088940)

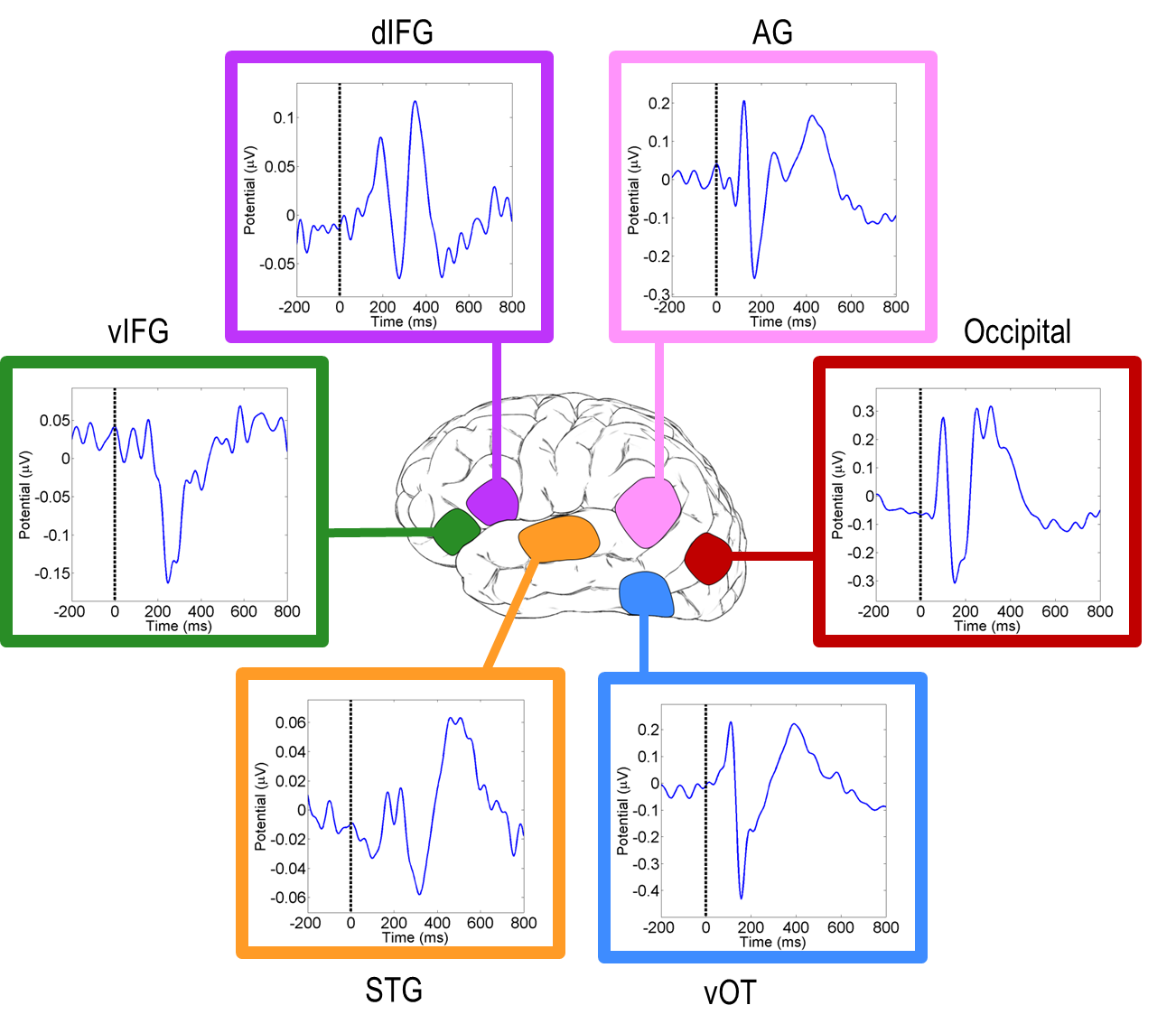

Supplement: Figure S1 — Dipole cluster ERPs. Area vOT shows a prominent N170 (negativity at ∼170 ms), a hallmark of categorical (and orthographic) processing. ERPs at other sites show similar, yet distinct ERP waveforms. vOT ventral occipito-temporal cortex; AG angular gyrus; STG superior temporal gyrus; IFG inferior frontal gyrus; d dorsal; v ventral. (TIF) [file pone.0088940.s001.tif]

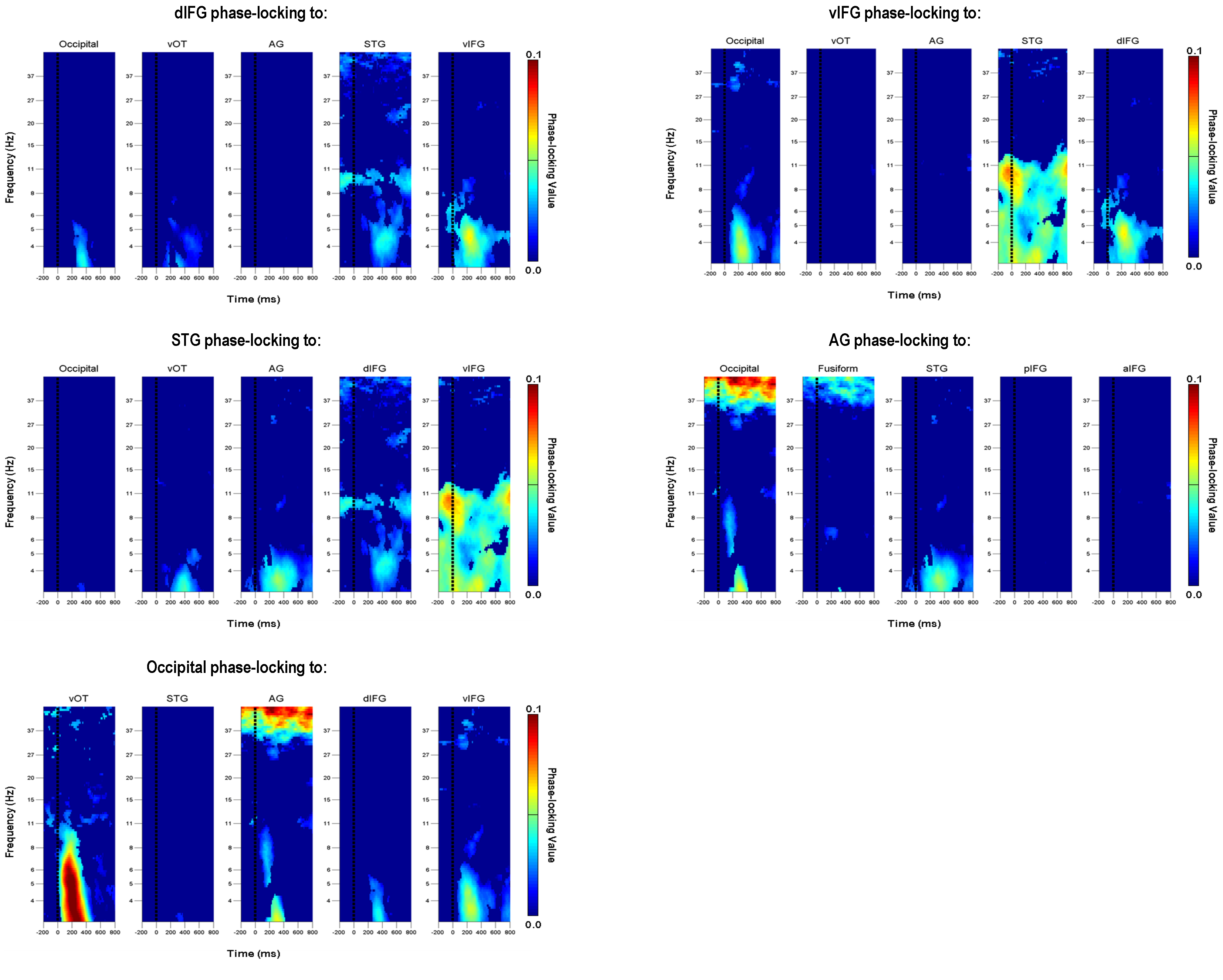

Supplement: Figure S2 — Time-frequency plots of PLVs among ROI IC clusters except for vOT with the others, which is shown in Figure 5a. For all phase synchrony analyses, individual subject PLV significance was computed at p = 0.001 by permutation test, and group significance was determined with a binomial probability of p = 0.000001. vOT ventral occipito-temporal cortex; AG angular gyrus; STG superior temporal gyrus; IFG inferior frontal gyrus; d dorsal; v ventral. (TIF) [file pone.0088940.s002.tif]

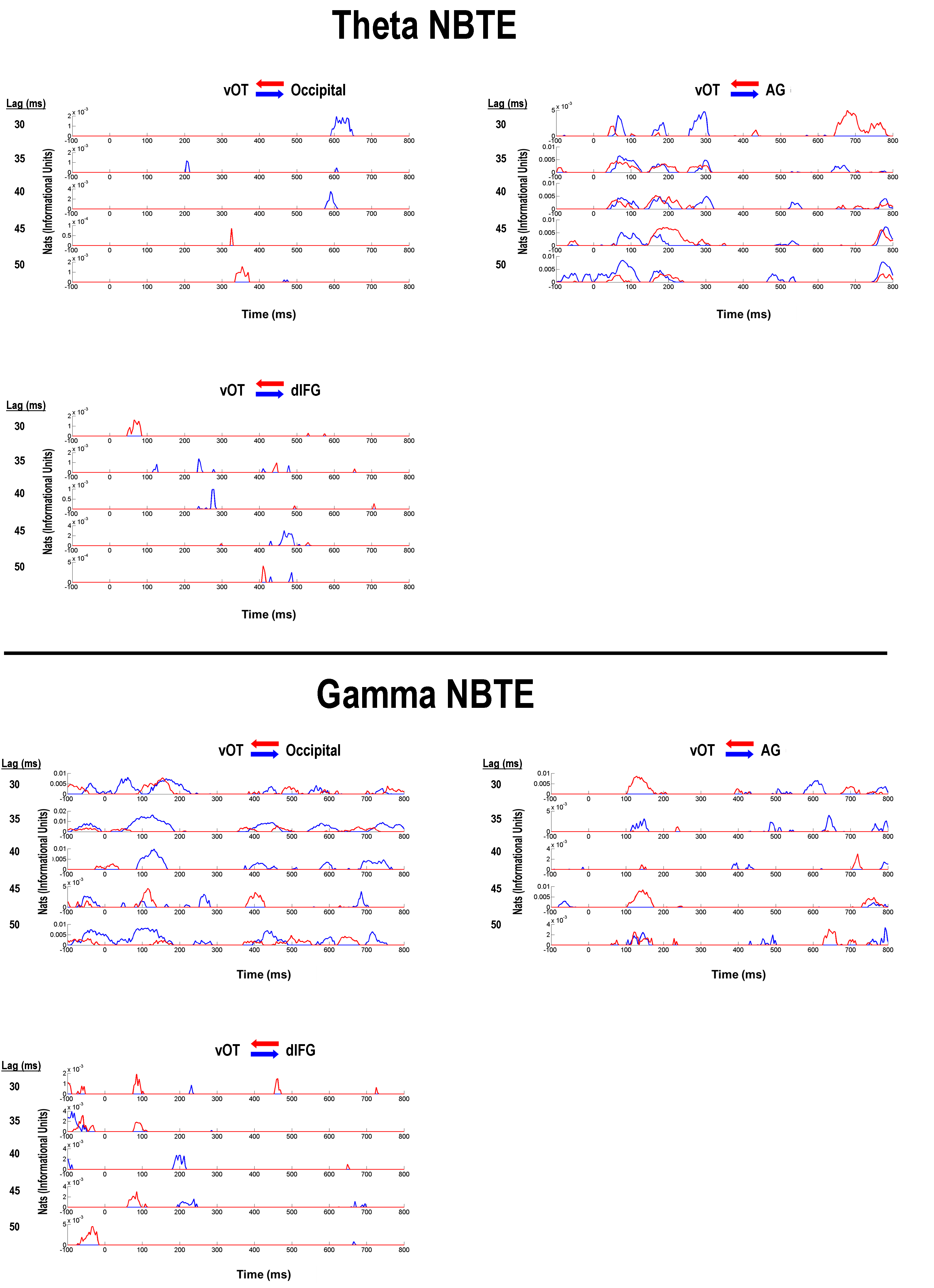

Supplement: Figure S3 — Example single-subject results from theta (3–7 Hz) and gamma (35–45 Hz) narrow-band transfer entropy (NBTE) analysis. Blue lines represent significant (by surrogate at p = 0.05) NBTE between vOT and occipital, AG, and dIFG regions, red lines represent the same from each respective site to vOT. Instances in which both NBTE patterns overlap signify a bi-directional relationship. Each small graph represents a different lag from 30 to 50 ms. (TIF) [file pone.0088940.s003.tif]
